# How do professionals and non-professionals respond to non-suicidal self-injury? Lived experiences of psychiatric outpatients in Singapore

**DOI:** 10.1186/s40359-023-01512-9

**Published:** 2024-01-04

**Authors:** Rachel Hsiao Shen Tan, Shazana Shahwan, Yunjue Zhang, Rajeswari Sambasivam, Say How Ong, Mythily Subramaniam

**Affiliations:** 1 Research Division, Institute of Mental Health, 10 Buangkok View, Singapore, 539747 Singapore; 2 Department of Developmental Psychiatry, Institute of Mental Health, 10 Buangkok View, Singapore, 539747 Singapore

**Keywords:** Non-suicidal self-injury, Help-seeking, Self-harm

## Abstract

**Background:**

For young people who engage in non-suicidal self-injury (NSSI), receiving negative responses to their NSSI can pose a barrier to future help-seeking. This qualitative study aimed to explore helpful and unhelpful ways in which professionals and non-professionals respond to NSSI, from the perspectives of individuals with lived experiences of NSSI.

**Methods:**

Semi-structured interviews were conducted with 20 outpatients (6 males, 14 females) aged 17 to 29 years from a tertiary psychiatric hospital in Singapore, who had reported engaging in NSSI behavior in an earlier study. The interviews were audio recorded and transcribed verbatim. Thematic analysis was used to identify themes and subthemes in the data.

**Results:**

Professionals’ responses were organized into three main themes: ‘prescribing solutions without understanding needs’, ‘disapproval or judgment’, and ‘helpful responses’. Non-professionals’ responses were organized into four main themes: ‘emotionally charged responses’, ‘avoidance and inaction’, ‘poor understanding of reasons for NSSI’, and ‘providing tangible support and acknowledging NSSI’. Participants also described how unhelpful responses negatively impacted their willingness to seek help.

**Conclusions:**

Our findings provide a better understanding of responses to NSSI that are considered helpful and unhelpful, and can be used to improve existing guidelines on responding to NSSI.

**Supplementary Information:**

The online version contains supplementary material available at 10.1186/s40359-023-01512-9.

## Introduction

Non-suicidal self-injury (NSSI) is defined as the “deliberate, self-inflicted destruction of body tissue without suicidal intent and for purposes not socially sanctioned” [1]. The most common form of NSSI is cutting or slashing, but individuals also engage in other behaviors such as burning, hitting, skin picking, and punching [2, 3]. The prevalence of NSSI in non-clinical samples ranges from 1.5 to 54.8% [3], while one study found that 58.8% of psychiatric outpatients between 14 and 35 years old engaged in self-harm [4]. NSSI is associated with significant emotional and psychological distress [5, 6], and may also be associated with suicidal thoughts and behaviors [5, 7]. NSSI can lead to further adverse outcomes which affect an individual’s wellbeing and functioning, including worsened psychological distress, higher rumination, more interpersonal stress, and poorer emotion regulation [8–10]. Help-seeking is thus important to ensure that individuals who self-injure receive the necessary intervention and support. NSSI behavior typically develops in adolescence, with an average age of onset of about 12 to 13 years old [11, 12]. However, between a third to half of adolescents do not seek help for their self-harm, either before or after a self-harm episode [13]. Hence, further research is necessary to examine factors relating to help-seeking for NSSI among young people.

Some facilitators of help-seeking for NSSI include assurances of confidentiality, having a trustworthy person to talk to, and knowledge of available options for help [13–15]. Conversely, barriers to help-seeking include stigma surrounding NSSI, experiences of shame, fears about receiving negative responses or being perceived as ‘attention-seeking’, beliefs that one could or should be able to cope alone, minimization of NSSI as a problem, and the belief that others would not understand the self-harming behavior [13, 15, 16].

Negative responses to NSSI which reinforce these fears and beliefs may deter individuals from future attempts at help-seeking [17]. For example, young people who turn to informal sources of help (e.g., parents, peers) often receive overly emotional responses such as anger, sadness, worry, shock, or confusion, and may consequently refrain from reaching out again to protect their loved ones from the intense emotions elicited by NSSI disclosures [18, 19]. Unsupportive or trivializing responses from parents cause reluctance in some young people to seek help again [19], while avoidant responses discourage future disclosure to other potential sources of support [16]. Similarly, patients seeking professional help for self-harm may disengage from services if they perceive staff to be dismissive or judgmental, or if their experiences with services leave them feeling hopeless [20]. On the other hand, individuals who receive positive and validating responses may be encouraged to continue seeking help, as having a caring environment and support from informal sources were found to be facilitators of professional help-seeking [14]. Helpful responses from non-professionals are those characterized by acceptance, remaining calm, and the provision of instrumental support [12, 16, 18, 19]. Clients also find it helpful when professionals respond nonjudgmentally and with genuine concern and understanding [20, 21].

Although prior studies have explored responses to disclosure of NSSI, few studies have specifically examined lived experiences of those who have self-injured, and often do not include responses from professionals. Singapore is a multi-ethnic country in Southeast Asia comprising a unique blend of cultures and religions which may influence attitudes and responses towards NSSI. For instance, in Chinese culture – where the concept of ‘face’ or reputation is highly valued – the stigma of mental illness extends to the family [22] and the act of NSSI is perceived to bring shame and disgrace to the family [15, 23]. Religious interpretations or lay folk beliefs about mental illness and the causes of NSSI (e.g., demonic possession, consequences of personal sin) may also add to negative attitudes towards individuals with NSSI [22, 24]. There is also a need to examine how both positive and negative responses to NSSI influence subsequent help-seeking attitudes and behaviors. Building on prior research, the present study sought to deepen our understanding of what constitutes helpful and unhelpful responses to NSSI in Singapore, linking these responses to future help-seeking.

The aim of this study was twofold: (i) to explore the responses of professionals and non-professionals towards NSSI, and (ii) understand how it relates to help-seeking behaviors for NSSI through in-depth interviews with young people with lived experiences of NSSI in Singapore. In addition, previous research has largely focused on reactions to disclosures of NSSI; however, in some instances, recipients may come to know of NSSI through discovery instead of disclosure, and it is equally important to include such instances when exploring lived experiences of reactions from others to NSSI. Hence, this study focused on responses to NSSI regardless of whether the NSSI was disclosed or discovered.

## Methods

### Participants

The study was conducted as part of a larger study comprising two phases. In Phase 1 of the study, a survey was administered to 400 outpatients of a tertiary psychiatric hospital to examine the prevalence and correlates of NSSI [4]. The Functional Assessment of Self-Mutilation (FASM) [25] was used to assess the frequency, functions, and other characteristics of participants’ NSSI within the past 12 months. Participants who were willing to participate in the second phase provided their contact information. Eligibility criteria for the second phase required participants to be between 14 and 35 years old, receiving outpatient psychiatric care, and have endorsed at least one of the NSSI acts listed in the FASM (e.g., ‘cut or carved your skin’, ‘hit yourself on purpose’). The final sample comprised 20 young people (6 males and 14 females) between the ages of 17 to 29 years. Participants were selected such that different genders, ethnicities, types and severities of self-injuries, and psychiatric diagnoses were well-represented in the sample. Table 1 presents demographic and clinical information of the sample.

**Table 1:** Participants’ demographic and clinical information

ID	Gender	Ethnicity	Diagnosis	Frequency of NSSI behaviours in the past 12 months (as assessed in the Functional Assessment of Self-Mutilation)										
				Cut/ carved	Hit	Pulled hair out	Gave self a tattoo	Picked wound	Burned skin	Inserted objects under nail or skin	Bit self	Picked skin to point of drawing blood	Scraped skin	Total NSSI
A1015	Female	Malay	Mood disorder	2	3	4	1	0	0	0	0	4	0	14
A1020	Female	Japanese	Borderline personality disorder	5	0	1	0	4	4	0	0	0	0	14
A1021	Female	Chinese	Schizophrenia	6	3	0	0	0	0	0	3	0	0	12
A1069	Male	Malay	Mood disorder	5	20	20	2	10	10	5	10	20	20	122
A1092	Male	Indian	Mood disorder	9	12	0	0	0	0	0	0	0	0	21
A2062	Female	Malay	Adjustment disorder	10	0	0	0	0	0	0	0	0	0	10
A2063	Female	Chinese	Mood disorder	5	2	0	0	3	0	0	0	0	0	10
A2072	Female	Indian	Adjustment disorder	3	0	0	0	0	0	0	0	3	0	6
A2090	Female	Chinese	Mood disorder	5	8	0	0	20	0	30	20	0	0	83
A3011	Male	Malay	Adjustment disorder	200	5	1	0	0	0	0	0	0	10	216
A3028	Male	Eurasian	Adjustment disorder	3	100	0	0	0	0	0	0	0	0	103
A3063	Female	Chinese	Anxiety disorder	20	50	100	0	20	0	2	5	5	5	207
A3080	Female	Chinese	Mood disorder	10	0	0	0	0	0	0	0	0	0	10
A4010	Male	Chinese	Adjustment disorder	9	0	0	0	2	0	0	0	0	0	11
A4021	Male	Chinese	Anxiety disorder	3	5	0	5	0	0	0	0	0	0	13
A4028	Female	Chinese	Mood disorder	20	5	0	0	0	0	0	0	0	0	25
A4052	Female	Chinese	Mood disorder	15	0	0	0	0	0	0	5	0	20	40
A4060	Female	Malay	Borderline personality disorder	30	10	1	1	30	2	0	2	5	5	86
A4073	Female	Chinese	Mood disorder	6	7	0	0	2	0	0	0	0	0	15
A4080	Female	Chinese	Mood disorder	100	10	0	0	200	5	0	0	2	0	317

### Procedures

Ethics approval for the study was obtained from the National Healthcare Group Domain Specific Review Board (DSRB No. 2014/01099), and all participants provided written informed consent. Semi-structured interviews were conducted between October 2015 and October 2016 by trained researchers, SS and MS. The interviews were held in interview rooms in the hospital’s research office. Each interview lasted about 60 min (ranging from 26 to 70 min) and was recorded using a digital audio-recording device, then transcribed verbatim by three of the authors (SS, YJZ, and RS). All participants were reimbursed $60 for their participation.

Participants were informed that the focus of the interviews was on NSSI behaviors. The present study was based on a subset of data elicited largely through the grand tour questions “Do you think NSSI has any impact on your life?” and “What are your thoughts about talking to someone about NSSI?”. Interviews were conducted until data saturation was attained.

### Data analysis

The data were analyzed using thematic analysis as outlined in Braun and Clarke’s six-step framework [26]. In Step 1, study team members (MS, SS, and YJZ) each read a subset of the transcripts line-by-line and coded inductively with an open coding approach. Reading and re-reading of transcripts allowed for familiarization with and immersion in the data. In Step 2, study team members compiled and organized initial codes into meaningful groups at a semantic level until a list of codes was agreed upon. This step was modified to include the creation of a codebook, which ensured that all coders had a shared understanding of the types of information to be grouped under a particular code. The codebook included the following information for each code: label, definition, inclusion and exclusion criteria, and examples of typical and atypical codes from the raw data. The codebook was reviewed by all team members and refined collaboratively. Using the preliminary codebook, all three study team members independently coded the same transcript to establish inter-rater agreement. NVivo11 was used to calculate the kappa coefficient between the three coders. Any major differences in coding were resolved; the coders repeated this process with a different transcript until a satisfactory kappa coefficient of 0.75 to 0.79 was attained for each pair. The 20 interview transcripts were then distributed among the three coders for independent coding using the finalized codebook. The results of this study are based on secondary data analysis of the transcripts, in which the authors (RT and MS) utilized a subset of the coded data (i.e., codes that addressed professionals’ and non-professionals’ responses to NSSI) to explore the aforementioned research questions.

In Step 3, the authors grouped similar codes to form potential themes, which involved the interpretation of data at a latent level. Coded extracts within each theme were collated. In Step 4, themes were gradually reviewed and refined in an iterative process to ensure that a concise, coherent, non-repetitive structure was attained. Codes which initially did not fit into any theme were revisited. In Step 5, the themes were presented by the authors (RT and MS) to the remaining co-authors for further refinement. Finally, in Step 6, the themes were finalized.

## Results

**Fig. 1:**
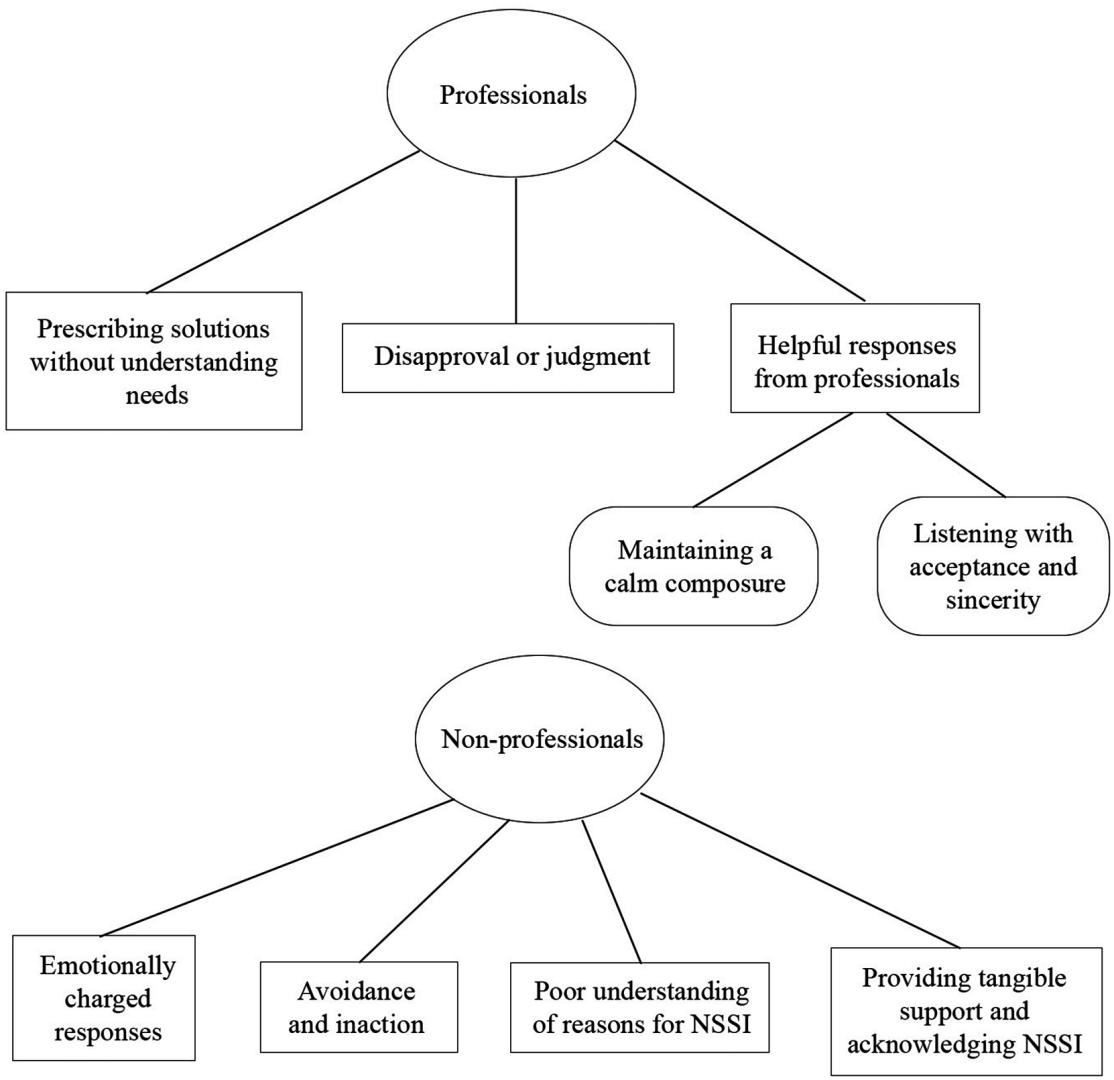
Themes and subthemes of responses to NSSI. Circles represent domains (i.e., professionals and non-professionals); Rectangles represent themes; Rounded rectangles represent sub-themes

Themes resulting from the analysis were organized into two overarching domains: responses from professionals and non-professionals that participants had experienced in response to their NSSI. Within each domain, we identified themes and subthemes that characterized the different ways in which professionals and non-professionals responded to the discovery or disclosure of NSSI, and its impact on participants’ help-seeking intentions. Professionals’ responses were organized into three main themes, while non-professionals’ responses were organized into four main themes (Fig. 1).

### Professionals’ responses to NSSI

Individuals with lived experiences of NSSI reported encounters with a variety of professionals, such as psychiatrists, nurses, general practitioners, and counsellors. These responses were organized into three major themes: prescribing solutions without understanding needs, disapproval or judgment, and helpful responses.

#### Prescribing solutions without understanding needs

Multiple participants (*n* = 10; 50%) spoke of occasions when professionals had offered interventions or solutions to NSSI that did not meet their needs. These included teaching psychotherapy techniques, prescription of medication, and applying standard procedures (e.g., warding the patient). On some occasions, participants reported feeling dismissed or misunderstood by care providers, and expressed frustration with professionals’ lack of understanding. These responses reflected an underlying need for professionals to first listen and understand the function of the NSSI before rushing to provide a solution.

*She asked me to think positively, but in the end, she doesn’t know that asking me to think positively is putting a lot of pressure [on me], and I am just afraid to come to XX hospital again. […] They keep telling me, “Never mind, it’s ok, you can do it, you can do it.” But for me, I was thinking, “Talk is easier than doing.” Because you’ve not been through what I have been going through.* (A1069)

Professionals who responded to NSSI in a rote manner without displaying genuine care were perceived by patients as being dismissive of patients’ emotional pain. Furthermore, recurring experiences with unhelpful treatment also caused frustration and disengagement from services. This is reflected in some of the verbatim presented below.

*“Oh, it’s okay, no problem. Ya, another 2 weeks I will see you.” To me it feels like I am not taken seriously. Then the moment I learn that they are not taking it seriously, the more I want to cut deeper. And show them.* (A1020)

*It makes me feel like (sigh) don’t want to seek treatment, you know like I see the same doctor, always the same ward […] then the doctor always says the same thing. Don’t want to help, the help is not helpful at all (breathes in) I don’t know what to do. So it frustrates me, it makes me not want to seek any treatment.* (A4080)

#### Disapproval or judgment

Responses from professionals that were characterized by harsh criticism, judgmental remarks, and disapproval surfaced prominently in a handful of accounts (*n* = 3; 15%). Participants described the emotional impact of these encounters as ‘hurtful’. Such responses caused some participants to recoil from treatment, while for others it exacerbated the urge to harm themselves. The fear of being looked down on by mental health professionals was stated by one participant as a reason for not disclosing NSSI. The following verbatim gives an example of some of the unhelpful comments made by professionals and illustrates the impact of these responses on participants.

*They will make comments like, “you’re being attention seeking”. […] Sometimes they have very disparaging remarks and it’s very hurting and it’s very triggering and the person would just go back and hurt themselves.* (A4080)

*My second or third visit wasn’t pleasant at all. It wasn’t, it makes me stop going to XX hospital. […] I went, when we entered in, the first question [the doctor] asked was, “How old are you?” I told him my age and he said, “Do you think [NSSI] is a responsible thing for you to do?” It was very hostile. I was crying.* (A1015)

#### Helpful responses from professionals

Participants also received responses from professionals which they found helpful (*n* = 10; 50%). These included maintaining a calm composure when treating NSSI, listening with acceptance and sincerity, and providing interventions that effectively met participants’ needs.

**Maintaining a Calm Composure.** As highlighted by a few participants, professionals who maintained a calm composure when reacting to NSSI were experienced as helpful. Such responses generally occurred in the context of receiving immediate medical attention for their NSSI wounds from doctors or nurses. A calm reaction, as opposed to one of high reactivity, seemed to convey an acceptance of the individual and their emotions. Furthermore, maintaining a calm composure, as opposed to catastrophizing the NSSI act, may also provide patients with a sense of comfort and reassurance.

*The [hospital staff] were quite natural like just, “okay so how are you feeling?” that kind of thing. Like normally, they didn’t have any big reaction. […] I was comfortable with it because it didn’t make me feel like I was weird.* (A3063)

*He dressed my wound. He seemed very calm and he didn’t seem like he was pretending like it was not there, but at the same time he was not doing things that made me feel embarrassed. […] He was very calming. And he was enough to make you feel like okay, things can move on, it’s okay.* (A1092)

**Listening with Acceptance and Sincerity.** Participants also expressed an appreciation for professionals who responded with acceptance and without condemning the person for their actions. In addition, professionals who made the effort to understand their patients’ needs by listening sincerely and displaying genuine care and concern were experienced as helpful. Participants who had the opportunity to relate their self-harm history to mental healthcare providers found relief in communicating their emotional distress. The participants described how being listened to nonjudgmentally was helpful to them.

*For some reason, [the counsellor] was very comfortable to speak to. Couldn’t explain it, you know, someone who could acknowledge what I was going through, who wouldn’t just dismiss it, wouldn’t just shout at me or label me as a psycho. I felt like she was someone who was accepting that I could finally open up my mind to speak to.* (A2072)

*[Interviewer: what was your experience like when you tell the doctors here about you harming yourself? Can you tell me how their reactions were and how satisfied you were with it?] Like finally someone can hear my story and hear my pain.* (A2090)

### Non-professionals’ responses to NSSI

Individuals with lived experiences of NSSI reported four types of encounters with a variety of non-professionals, such as parents, extended family members, friends, and significant others that are described below. These responses were organized into four major themes: emotionally charged responses, avoidance and inaction, poor understanding of reasons for NSSI, and providing tangible support and acknowledging NSSI.

#### Emotionally charged responses

Non-professionals’ immediate reactions to finding out about participants’ NSSI were typically characterized by high reactivity and intense emotions, including sadness, anger, shock, and fear (*n* = 11; 55%). The intensity of the responses was oftentimes uncomfortable or overwhelming for participants. Furthermore, emotionally charged reactions can cause the individual to feel burdened or guilty about the impact of their NSSI on their loved ones. The impact of intense emotional responses on participants is reflected in the verbatim below:

*My hand got cut, then it was a lot of blood, then my dad got scared and sent me to the hospital, but while sending me to hospital, he was cursing [at] me.* (A1069)

*For my dad and my brother I was quite upset because I felt like it was my fault for making them feel like worried, concerned, and then my brother even cried ‘cause he hardly ever cries. So I felt that if I didn’t do anything, they wouldn’t have to feel this way.* (A3063)

#### Avoidance and inaction

Another common response from non-professionals was that of avoiding or distancing themselves from the individual who self-injured (*n* = 10; 50%). Participants described experiences of being ostracized by peers or family members who found out about their NSSI. Some participants had turned to loved ones to disclose NSSI in search of social support, but were met with the opposite effect, as non-professionals who distanced themselves from participants may have added to existing feelings of isolation and loneliness.

*I guess family members, a lot of people just don’t want to associate with me now because I’m not a positive influence. Right, I’m cutting myself and that’s a sign of mental illness already. So people are just avoiding me more now.* (A2072)

*I felt that if [my school mates] tried to help and they didn’t see me visibly getting any happier, then they probably thought they are making it worse, then they would just stop talking to me. So I guess that was the worst. Just felt much more alone.* (A3011)

On a related note, some participants also spoke of incidents when family or friends had evidently noticed their NSSI but intentionally chose to avoid any discussion on the topic. Ignoring visible NSSI marks was perceived as a lack of concern, and some participants expressed frustration with the fact that their distress had gone unnoticed by those around them:

*They didn’t usually talk to me about it. Like no one so far has talked to me about it. The most they just say don’t do it again.* (A4060)

*No one really noticed it. Even if I’m dead or have marks, they don’t ask. They didn’t even ask anything. Even my parents saw it, they didn’t say anything. [I: And how do you feel about that?] Like, “what am I going to show you to tell you that I am in pain?”* (A2090)

#### Poor understanding of reasons for NSSI

Many non-professionals had difficulty grasping the reasons or intentions for which their loved ones would intentionally harm themselves (*n* = 9; 45%). This was reflected across multiple accounts in which family and friends questioned participants about the reason for their NSSI, often in an accusatory and interrogative manner. Participants described the struggle of facing loved ones who could not grasp the reasons for their NSSI. This is reflected in the following quotes:

*[My parents] don’t know the struggle I go through. They think it is easy to stop, it is not. I wish they would understand what is going through my head.* (A1020)

*I wish they would understand why I do it and why I continue to do it instead of asking me not to do it.* (A4073)

A poor understanding of reasons for NSSI also led to uncompassionate responses from non-professionals. Some participants experienced blatant judgment from others in response to their NSSI and were labelled with harsh words like “crazy”, “stupid”, and “selfish”. Responses from non-professionals also conveyed a sense of disapproval of participants’ actions. In some cases, the judgmental attitudes of non-professionals towards NSSI in general was sufficient to discourage participants from disclosing their own NSSI. Below are a few examples of the harsh responses received by participants and the effects of such comments on the person.

*They told me that I just stupid. Like why do you do this to yourself? Why do you harm yourself?* (A2062)

*If I told them, they would have labelled me as crazy. I remembered one time in my [co-curricular activity group], we got on the topic about people self-harming. There’s this girl saying, “Those people are retarded. Why would they do such a thing?” So it furthered my stand of not telling anybody.* (A3063)

#### Providing tangible support and acknowledging NSSI

Nearly half the participants shared about positive encounters with non-professionals who had responded in a compassionate and supportive manner (*n* = 9; 45%). Helpful responses included checking in with the person, spending time with them, and providing a listening ear. Other responses directly addressed the NSSI behaviour, such as encouraging the individual to refrain from further NSSI out of concern or offering advice for wound care. Participants described some supportive responses from non-professionals as follows:

*She’s very kind, she sometimes [invites me over to] her house. Like, whenever she is available she says, “Okay you can come to my house to do your work today”. Stuff like that.* (A1020)

*My friend who had depression, he told me that like if you really feel like cutting and you just can’t do anything about it, you might as well do it properly and just be safe. Use a cream, get a first aid kit, just clean up the wounds properly so that it won’t get infected.* (A3011)

Furthermore, acknowledging the NSSI – instead of ignoring it – provides the person with the opportunity to have their pain validated. Participants expressed a sense of relief at opportunities to have their pain seen and validated. A participant, whose classmates had noticed her self-harm marks and approached her out of concern, appreciated the way her peers had acknowledged and paid attention to her struggle:

*To me like after two years of like being in the same class, they finally show some concern and care about me, that makes me feel appreciated.* (A2062)

A common pattern observed across these accounts was that the support provided by non-professionals rarely addressed the emotional distress driving the NSSI behaviour. Despite this, participants attested that the provision of social support in various ways was sufficient to make them feel cared for, and this was experienced as helpful. For example, a participant, who initially started engaging in NSSI after breaking up with his girlfriend, described how social support from a friend was helpful despite not directly addressing the reason for his NSSI:

*I guess what I really wanted was a sense of companionship ‘cause I didn’t really get any love from my family and when I broke up with my girlfriend, it just felt more empty. So I guess in a way, he couldn’t have helped in that part. But at least him being there, talking to me, hanging out just felt like at least someone cares.* (A3011)

## Discussion

Our study identified different ways in which professionals and non-professionals respond to NSSI, organized into three main themes from professionals and four main themes from non-professionals. While some of these responses were perceived as helpful, more were perceived as unhelpful by the young people with a history of NSSI. Overall, we also observed that responses to NSSI commonly impacted participants’ willingness to seek help subsequently. Negative responses from professionals not only disrupted the therapeutic alliance in some cases but also caused young people to disengage from treatment. Similarly, some participants who encountered unhelpful responses from non-professionals were discouraged from reaching out for support again. Therefore, unhelpful responses to NSSI can significantly alter the trajectory of help-seeking behaviors.

### Unhelpful responses to NSSI

Broadly, we observed that perceived unhelpful responses – across professionals and non-professionals – were characterized by the effect they had on the person with NSSI. These were responses which made young people feel alienated, misunderstood, ashamed, or dismissed. A common unhelpful response from professionals was the prescription of solutions which inadvertently overlooked the person’s emotional needs. Clinicians may sometimes feel a need to “rescue” the client who has self-harmed, and end up rushing to fix the behavior with stopgap interventions which are ultimately unhelpful for clients [27]. Additionally, professionals who provided services in a rote manner were perceived as being dismissive of emotional pain and not taking the patient’s distress seriously. Patients with self-harm behaviors understandably desire to be treated by clinicians first and foremost as a person, instead of a medical case or a textbook example [28].

The young people in our study experienced being avoided or ostracized by non-professionals in response to their NSSI, which added to feelings of stigmatization and shame. Participants also shared that non-professionals often took no action after noticing visible NSSI marks and this was perceived as unhelpful. Non-professionals who suspect that their friend or family member is self-injuring may adopt a “wait and see” approach in hopes that the situation will resolve itself [29], and family members may hesitate to broach the topic due to uncertainty about how to address it or fears that it could trigger another NSSI episode [18]. However, such avoidance and hesitation can inadvertently convey to individuals with NSSI that their distress is not significant enough to elicit concern from loved ones. Given that one of the functions of NSSI is to communicate extreme distress and elicit concern from others [30], responses that ignore or dismiss NSSI are understandably unhelpful for those who engage in it as a cry for help.

Other unhelpful responses included those which conveyed disapproval and judgment of the young person. In line with existing research, participants reported that perceived judgment from professionals reinforced feelings of shame, causing subsequent avoidance of professional help-seeking [31, 32]. Likewise, responses that conveyed a poor understanding of the reasons for NSSI were common among non-professionals and often evoked extreme and unkind responses. Many participants faced harsh judgment, disapproval, or labelling from the people around them in response to their NSSI. This is consistent with findings that people who have misconceptions about the functions of NSSI are more likely to endorse negative responses such as ignoring the person [33]. We observed that the judgment and disapproval expressed by others, particularly those in close proximity to the individual, may intensify feelings of shame and perceived stigma, causing the person to shy away from future help-seeking attempts. Previous research similarly found that parents were unable to fully understand their child’s self-harm and their instinctive response was to question their child’s motivations for self-harm [29]. However, such responses can cause further self-degradation and worsened self-injury [28].

One reaction which was unique to non-professionals was that of responding with extreme emotional reactions. This is consistent with previous research, both from the perspectives of people with lived experiences of NSSI [19] as well as non-professionals who responded to NSSI [34, 35]. Variations in responses from professionals and non-professionals may be due to differences in psychological proximity to the individual engaging in NSSI [28]. Non-professionals, especially family and close friends or significant others who have closer emotional proximity to the individual with NSSI, may be unable to fully understand and accept the person’s NSSI which causes them undue worry and distress [36]. The knowledge that a loved one has been self-injuring can be devastating and the intensity of emotions often stems from a place of concern [29, 35]. Nonetheless, overly emotional responses are experienced by young people as uncomfortable and could intensify distress or exacerbate the desire to self-injure [18]. Furthermore, emotion-laden responses may cause people to refrain from reaching out to loved ones for support in the future to protect them from feeling upset [28]. Additionally, unhelpful responses from peers and family members were situated within the contexts of interpersonal relationships and tended to be especially harmful due to repercussions for the relationship. While informal sources of support can be extremely valuable for people with mental health struggles due to the accessibility and continuous nature of support (as compared to professional help which is usually time-limited) [37], negative changes such as ruptures in the relationship or stark changes in family dynamics are also felt more strongly [17, 18, 38].

### Helpful responses to NSSI

On the other hand, helpful responses across both domains were distinctly characterized by an acceptance of the individual and their NSSI behavior, without overreacting to it. Participants highlighted several responses from professionals which were experienced positively. Firstly, professionals who maintained a calm and assuring disposition when treating NSSI wounds were perceived as helpful. This corroborates Wadman and colleagues’ observation that youths prefer responses of “understated acceptance” instead of overtly emotional reactions [19]. Secondly, participants also found it helpful when professionals provided an opportunity to express their emotional pain and responded with acceptance and sincerity. This corresponds with existing research which shows that individuals with NSSI found it helpful when recipients of disclosure were able to accept the fact that they were engaging in NSSI without condemning or questioning their actions, and that responses which convey acceptance of the individual can lessen feelings of shame [16]. Similar to previous studies, we also found that professionals who conveyed genuine care and concern for the patient were experienced positively [21, 39].

Helpful responses from non-professionals typically involved reaching out to the person with NSSI in an empathetic and accepting manner. Our results suggest that helpful support does not necessarily need to address the root cause of NSSI directly. Instead, participants appreciated non-professionals who simply made the effort to be present and provide tangible support. Empathetic listening was also identified as a helpful response. These results align with previous findings that tangible aid and emotional support were both perceived as helpful responses to NSSI disclosure [40].

### Implications

Collectively, these results provide deeper insight into how professionals in different settings can better respond to and support individuals seeking help for NSSI. These findings underscore the importance of building and maintaining a strong therapeutic alliance with self-injuring patients, characterized by genuine care, trust, and nonjudgmental acceptance [21, 41]. Importantly, our results also emphasize the need for professionals to find the delicate balance between ‘not catastrophizing’ (i.e., avoiding overly emotional reactions) and ‘not minimizing’ (i.e., not dismissing patients’ emotional pain) the acts of NSSI by young people. These findings corroborate existing guidelines that encourage professionals to maintain a calm, caring, and compassionate demeanor, and to avoid over- or under-reacting to NSSI behavior [42].

Our findings also highlight the need to further educate both professionals and the public on NSSI. Programs which equip professionals and caregivers with knowledge about NSSI can increase NSSI literacy and empower them to respond in more empathetic and accepting ways [43]. Furthermore, specific training on what constitutes helpful and unhelpful responses to NSSI will be beneficial for professionals and non-professionals supporting individuals who engage in NSSI. Mental health literacy programs in school can incorporate materials to equip youths with practical knowledge about how to respond appropriately if they learn that a peer is engaging in NSSI. Lastly, psychoeducation that addresses possible reasons for NSSI may also help to improve non-professionals’ understanding of the behavior, and improve attitudes and decrease stigma towards individuals who engage in NSSI [44].

### Limitations

There are several limitations of this study. Firstly, due to the small sample size, findings may not be generalizable to the broader population of individuals with NSSI behaviors. Similarly, participants who agreed to be interviewed may have had differing experiences from those who declined (e.g., the latter group may have had more positive or negative encounters with disclosure, or differing severity of NSSI). Next, all participants in our sample were receiving treatment for a mental health condition; therefore, our results may not capture the unique experiences of individuals with NSSI who have never sought help for their mental health struggles. Additionally, participants in our study had not necessarily sought help for their NSSI behavior specifically; their experiences might not be representative of individuals who had entered mental health treatment due to NSSI. Lastly, due to the retrospective nature of participants’ accounts, the experiences shared by participants would be subject to potential errors or biases in recall.

### Conclusion

The aim of the current article was to provide an understanding of responses to NSSI that are considered helpful and unhelpful by individuals with lived experiences of NSSI. Participants reported a range of helpful and unhelpful responses from professionals and non-professionals and shared about the impact of these responses on their future willingness to seek help for NSSI. These findings provide valuable insight on how to better support young people in their recovery and help-seeking process for NSSI.

### Electronic supplementary material

Below is the link to the electronic supplementary material.

**Supplementary Material 1**: Interview Guide

## Data Availability

Participants of this study did not agree for their data to be shared publicly. Supporting data is therefore not available.

## Abbreviations

FASM: Functional Assessment of Self-Mutilation
NSSI: Non-suicidal self-injury
