# Supplementary material for: How do professionals and non-professionals respond to non-suicidal self-injury? Lived experiences of psychiatric outpatients in Singapore
Source: BMC Psychol. 2024 Jan 4;12:14. doi: 10.1186/s40359-023-01512-9 (PMC10768356; doi:10.1186/s40359-023-01512-9)
Supplement: Supplementary file 1 — Supplementary Material 1: Interview Guide [file 40359_2023_1512_MOESM1_ESM.docx]

**Supplementary File 1 (Interview Questions)**

Consequences

Do you think NSSI has any impact on your life?

Does your NSSI have any influence on your relationships?

(e.g. schoolmates, friends, colleagues or family members, romantic partners)

What other impact has it had?

Help-seeking and barriers

*(Traditional social support)*

What are your thoughts about talking to someone about NSSI?

Have you talked about NSSI to someone?

How comfortable do you feel talking about it?

Has anyone ever found out about your NSSI? (e.g. parents, healthcare professional, teacher or counselor?)

How helpful/unhelpful were they?
